# Influence of Complexity and Gestalt Principles on Aesthetic Preferences for Building Façades: An Eye Tracking Study

**DOI:** 10.16910/jemr.17.2.4

**Published:** 2024-08-09

**Authors:** Dilara Beder, Matthew Pelowski, Çağrı Imamoğlu

**Affiliations:** Bilkent University, Turkey; University of Vienna, Austria

**Keywords:** Eye tracking, eye movement, visual perception, complexity, Gestalt, aesthetic preferences, façade design, architecture

## Abstract

Buildings are an integral part of our physical environment and have aesthetic significance with respect to the organizational integrity of architectural elements. While Gestalt principles are essential in design education, their relationship with architectural features remains understudied. The present study explored how Gestalt principles and complexity levels influence evaluations of building façades through the use of questionnaires and eye tracking. Twenty-four two-dimensional black and white façade drawings, manipulated using selected Gestalt principles (similarity and proximity) to achieve different levels of complexity (low, medium & high), were presented to 79 participants. The results suggested a negative linear relationship between aesthetic ratings and complexity levels across selected Gestalt principles. In addition, as expected, participants had the highest number of fixations, shortest fixation durations, and lowest aesthetic ratings for higher levels of complexity. Results involving Gestalt principles revealed that proximity-based designs received higher aesthetic ratings, demanded less time, elicited lower number of fixations, and resulted in shorter fixation durations. Conversely, similarity-based designs received lower aesthetic ratings, demanded more time, elicited higher number of fixations, and resulted in longer fixation durations. These findings offer insights into architectural aesthetic experiences and inform future research directions.

## Introduction

Aesthetics is a complex phenomenon that is associated with beauty (37), pleasure (30), liking or appreciation (17). Fundamentally, explaining the nature of aesthetic experience is an important topic in psychology (44). Aesthetic appeal is associated with an individual’s attention to objects, as people look longer at things they find beautiful or attractive (53), works of art (52; 75), or their physical environment (4; 79). Aesthetic experience is defined as a process of integration of sensory information, cognitive processes, but also evolutionary responses. Therefore, the visual system plays an important role in interpreting and making sense of the stimuli around us. When people encounter an object or work of art, their visual perception goes through a complex interaction that involves the analysis of many elements such as shapes, colors, textures and spatial relationships (15; 26). Complexity has been regarded as an important feature in aesthetic evaluation (9). Although the definition of complexity may depend on the specific field of study, it is generally interpreted as the difficulty of understanding the whole with countless parts. Furthermore, it is argued that the importance of complexity in our perception is well derived from the development of Gestalt psychology (20). This is because the human cognitive system may more effectively comprehend the complexity of a composition when it is organized in a structured manner. Visual perception, that is, the information that reaches the eye, is followed by the processing and organizing of that information (24; 61; 64).

The connection between individuals and their living environments, as well as their aesthetic assessments of those surroundings, such as building façades, presents ample research opportunities. In empirical aesthetics, architectural façade organizations have been studied (32; 47). Architectural organizations and people’s aesthetic evaluations have generally been studied in relation to compositions at different levels of complexity (2; 33; 34; 38). However, the interaction of organizational elements of Gestalt principles and complexity levels on subjective evaluations of building façades has not yet been investigated. Therefore, the first aim of the present study was to explore how the stimulus organization obtained by Gestalt principles interacts with people’s aesthetic evaluations at different complexity levels for building façades. There are many studies (25; 62; 65; 67; 81) about how people perceive and evaluate their environment using subjective methods. However, perception is a voluntary activity (5) and there has been little quantitative analysis using both the subjective and objective evaluations of participants. Motivated by the above considerations, the second aim of the study was to analyze the participants' subjective and objective evaluations together in a perception study. Therefore, in addition to using a questionnaire to obtain subjective evaluations from participants, the present study also used an eye-tracking system to obtain objective evaluations of participants from their visual scanning behavior.

## Literature Review

### Complexity, Gestalt and Aesthetic Evaluations

Complexity was found to be a significant influence on aesthetic evaluation (12; 34; 36; 69). Birkoff (10) expresses the relationship between aesthetics and complexity with a mathematical formula. He argues that aesthetic ratings tend to be positively correlated with order, while being negatively correlated with complexity. According to his approach, organized and simple objects have the highest aesthetic value, and many studies have supported this linear relationship (10; 12; 27; 69). In contrast to the approach of Birkoff, Berlyne (8) defines complexity as irregularity of shape and suggests that there is an inverted U-shaped relationship between hedonic experience and complexity. Accordingly, stimuli with a medium level of complexity would be preferred more than the others. Numerous studies, such as studies of urban landscape visual material (38), architectural building ( 34; 55) and façade illustrations (55), support Berlyne’s hypothesis.

As the complexity of the environment increases, there is a greater need to simplify and condense incoming information to effectively comprehend and orient ourselves (74). In terms of perceiving the physical environment, the control of complexity is the basis of Gestalt theory (20) which is concerned with the spontaneous organization of the human brain in the physical environment (39). By understanding the natural structure of information in our minds, researchers have gained insights into cognitive processes and human behavior. The development of the Gestalt psychology movement can be seen as a reaction to the then dominant reductionist approach which sought to understand the mind by dissecting it into its component parts (39). In short, the theory posits that when people are exposed to unfamiliar visual information, the mind organizes the data by focusing on the form as a whole, rather than its component parts (14; 39; 80). The theory aims to explain how people arrange visual elements to form a whole, employing grouping principles. These principles, known as Prägnanz, are also referred to as the Gestalt principles of visual organization.

Aesthetic evaluation is a crucial concept in design disciplines such as architecture, interior design and visual design, and Gestalt theory is a central theme in studies examining the relationship between perception and aesthetic evaluation. Although the number of experimental studies explaining the effect of Gestalt principles on aesthetic evaluation is limited, it has been shown that images with similarity properties have lower beauty ratings than those with closure, proximity, and figure-ground properties (14). People spontaneously perceive information about their physical environment and transform it into aesthetic-evaluative outcomes through eye-brain cooperation (S. 46; 79). The Bauhaus masters stated that ‘dots’ are the foundation of design and these dots combine to create lines, textures and volumes (42). The Bauhaus master's basic design theory is based on the Gestalt theory of perception. According to this, the aesthetics of architecture is emphasized by the principles of the organization of architectural forms and the propositions of their elements (41); that is, the basic elements of geometry follow the compositional organizations. Architectural experience is influenced by the form of the physical environment (79), and aesthetics in architecture is constructed by the geometric organization of elements that are evaluated by architects or interior designers guided by Gestalt theory (41). People cannot perceive their physical environment in a random order, and Gestalt theory supports the organization of elements (72). If the forms are organized according to Gestalt law, they are referred to as ‘good forms’, but this is not precisely an evaluative statement (42), as there is no evidence in Gestalt theory to suggest that good and attractive are equivalent (43). In addition, Arnheim (3) defined the Gestalt approach to architectural form and showed that it could be analyzed on the basis of symmetry, size, quantity and location of forms.

### Aesthetic Evaluations and Eye Tracking

Eye movements have been regarded as an integral and essential part of visual perception (57). We can obtain the brain's high-level unconscious brain activity processes and visual perception by observing eye responses (61). For this reason, eye-tracking systems are utilized to investigate the eye movement behavior of individuals, potentially elucidating underlying brain activity (56; 64).

Architectural works are complex physical structures both in two and three dimensions. Accordingly, in recent years, eye-tracking systems have been used to analyze the perceptual evaluations of physical spaces (16; 18; 22; 15; 48; 71; 73; 79), the effect of three-dimensional environments on liking (46), environmental complexity on aesthetic evaluations (63), and the effect of balance of façade compositions on aesthetic judgment (32). In this regard, eye metrics of fixations and saccades have been seen as the main measures of exploring individuals’ engagement with and perception of the visual world (7). Visual perception processes occur during a fixation ( 40; 49). Number of fixations refers to the frequency of occurrence of a particular state, while fixation duration states the duration of time during which the eye remains motionless while looking at a specific region, measured in milliseconds (14). Fixations are the intervals that occur between saccades (synchronous movement of the eyes from point to point while scanning the visual field), during which the eyes remain relatively stationary (59).

Experimental evaluation on visual search points to the effect of stimuli complexity on eye movements; for example, in related studies, complexity of images was determined based on fixation and saccade gaze points, such that the number of fixations increased (31; 77), trial duration increased ( 11; 13; 77) and fixation duration decreased (29; 35) during exposure to complex stimuli. Based on the findings from empirical aesthetics, it is suggested that visual fixations play a role in aesthetic evaluations and fixation duration, number of fixations, and total viewing duration, are significantly correlated with aesthetic ratings (78). Longer fixation durations with lower number of fixations are associated with higher aesthetic ratings (50). Furthermore, in a study of Gestalt principles on photographic compositions, participants had the greatest number of fixations and the lowest aesthetic ratings in response to photographs organized using similarity compared to closure, figure-ground and proximity (14).

### Research Questions and Hypotheses of the Study

As can be inferred from the studies mentioned above, the effect of visual organization obtained by Gestalt principles on aesthetic evaluation (to our knowledge) has not yet been investigated from an architectural point of view. Architectural organizations and people’s aesthetic evaluations have generally been studied in relation to compositions at different levels of complexity (19; 28; 32; 34; 38). However, the interaction of organizational elements of Gestalt principles and complexity levels on objective evaluations in architectural studies remain an open question. For this reason, we posed two research questions and hypotheses to achieve the objective of our study, which are detailed below.

Research Question 1: How are Gestalt principles and participants’ aesthetic evaluations of façade compositions related at different levels of complexity?

Hypothesis 1a: In line with the findings which associate simple and well-organized objects with highest aesthetic values (10; 12; 27; 69), we hypothesized that aesthetic rating of façade compositions would be negatively correlated with their complexity levels.

Hypothesis 1b: Based on the past literature (14) we hypothesized that façade compositions designed with the manipulation of the similarity principle would have lower aesthetic ratings than the ones designed with the manipulation of the proximity principle.

Research Question 2: How do the effects of Gestalt principles and levels of complexity influence participants’ eye metrics in response to façade compositions?

Studies describe the role of visual fixations in defining visual complexity and aesthetic evaluations (11; 13; 29; 31; 35; 77). In those studies, visual fixation data are characterized by fixation duration, number of fixations, and total viewing time.

Hypothesis 2a: As visual complexity increases, aesthetic evaluation and, consequently, fixation duration decrease (29; 35; 50). Thus, we hypothesized that participants would have the lowest fixation duration scores at high complexity levels.

Hypothesis 2b: Past studies indicate that as visual complexity increases, aesthetic evaluation decreases, and consequently, the number of fixations increases (31; 50; 77). Accordingly, we hypothesized that participants would have the highest number of fixations at high complexity levels.

Hypothesis 2c: In line with past reports stating that as visual complexity increases, aesthetic evaluation decreases, and consequently, total viewing time increases (11; 13; 77), we expected that participants would have the highest total viewing duration scores at high complexity levels.

To address our aims and test our hypotheses, we investigated participants’ aesthetic evaluations and gaze metrics in response to façade compositions designed with the manipulation of the selected Gestalt principles (similarity and proximity).

## Methods

### Participants

A total of 79 participants (49 female, 30 male; *M*_age_ = 27.92, *SD*_age_ = 2.90, range: 25–32 years) were included in the analyses (11 participants were excluded from the study due to unstable eye movements). All participants included in the analysis had normal or corrected-to-normal vision. The study corresponds with the ethical standards of the Declaration of Helsinki and the ethical regulations at University of Vienna and Bilkent University. 

### Visual Stimuli

The instruments have been designed by two designers with graduate degrees in architecture. Within the scope of the study, two Gestalt principles suitable for manipulation in 2-dimensional façade design, namely similarity and proximity, were selected (54). Additionally, in light of previous studies (33; 45), additional elements that would give the façade a different identity, such as ornamentation, were not included in the façade drawings. For this reason, in our study, we utilized the same number of elements at all levels of complexity. While color seems to have an impact on our cognition, affect and behavior, it may also influence our aesthetic preferences (21; 66). Thus, to reduce the potential influence of color and individual preference (1), the stimuli were produced in black and white.

**Figure 1.**
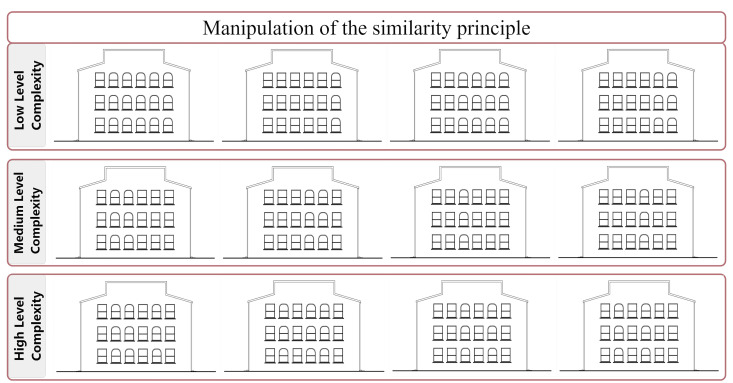
Building façade drawings manipulated according to the similarity principle

**Figure 2.**
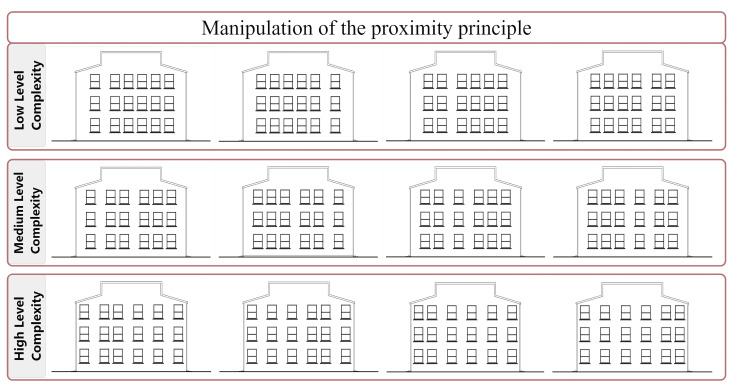
Building façade drawings manipulated according to the proximity principle

To control for familiarity, a group of drawings was produced using photographs of existing buildings in Vienna, since all participants were residents of the city. After examining the building façades, we chose windows with either orthogonal or circular designs. Observing that most buildings were 3 to 5 stories high, we standardized the façade drawings to a 3-story structure to reduce complexity. After the initial images to be used in the study were created, 15 architects, serving as experts, assessed the complexity levels of 36 manipulated façade drawings. These drawings were divided equally, with 18 manipulated according to the similarity principle and the other 18 according to the proximity principle. The architects rated the complexity of each drawing using a 7-point Likert scale with bipolar adjectives, ‘simple – complex’. Following the ratings, the distributions of the complexity ratings for each drawing were examined, and 12 drawings that did not exhibit a normal distribution were not included in the main study. A total of 24 façade drawings were used in the main study, 12 of which were manipulated according to the similarity principle and the remaining 12 according to the proximity principle (see Figures 1 & 2). The façade drawings in both groups were divided into three subgroups based on the mean complexity ratings: low complexity (consisting of images with average ratings of 1 to 4), medium complexity (consisting of images with average ratings of 5 to 8), and high complexity (consisting of images with average ratings of 9 to 12).

**Figure 3.**
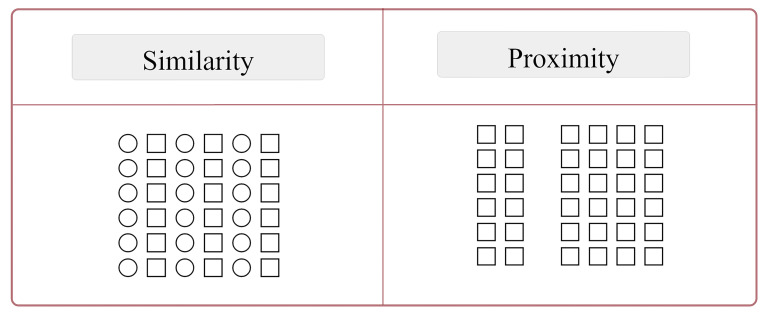
Illustrations of Gestalt principles of similarity and proximity (adapted from 82)

**Figure 4.**
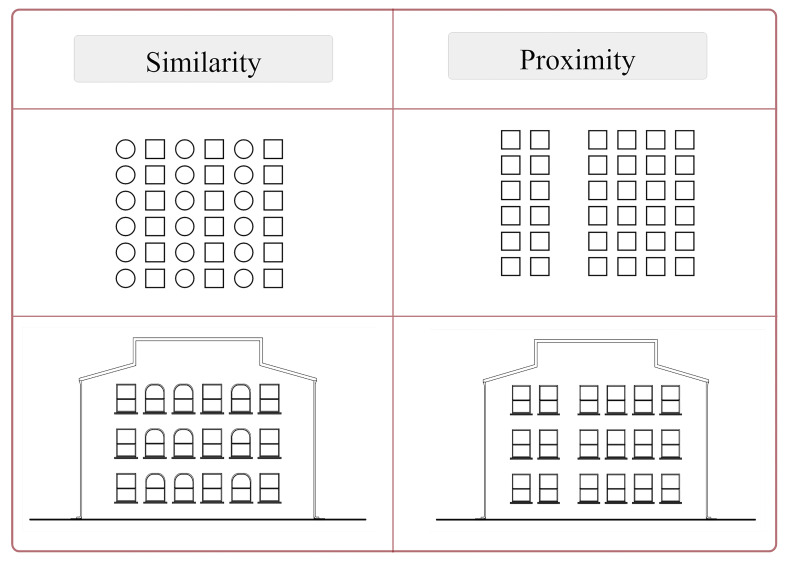
An example of illustrations of the present study demonstrating the manipulation of stimuli in accordance with Gestalt principles of similarity and proximity (adapted from 82)

The principles of Gestalt theory that are relevant to our research will be presented below (see Figures 3 & 4).

**(i) Similarity:** As per the similarity principle, parts that exhibit similar characteristics are perceptually grouped together (see Figure 3; 58); in the present study, the similarity principle was manipulated according to the shape differences of the windows (see Figure 4). As more variations in geometric differences (orthogonal vs circular) are introduced to the window frame groups, the complexity levels of façades increase (see Figure 1).

**(ii) Proximity:** According to the proximity principle, people tend to perceive those objects that are close to each other as part of a group (see Figure 3; 80). Thus, in the present study, we manipulated this principle by altering the distances between the windows (see Figure 4). As more variations in distance differences are introduced to the window frame groups, the complexity levels of the façade increase (see Figure 2).

### Questionnaire

The survey data consisted of two parts. The first part comprised a 7-point Likert scale bipolar adjective question, ‘simple-complex’ designed to measure the level of complexity perceived by the participants for each stimulus. The perceived complexity ratings were used to verify that participants perceived the level of complexity of the drawings as manipulated (see Results section). The second part consisted of participants’ evaluations of each stimulus using 7-point Likert scales in terms of the following bipolar adjectives: unpleasant-pleasant; distressing-relaxing; meaningless-meaningful; chaotic-coherent; commonplace-novel; unclear-clear. Survey questions were prepared with reference to previous studies (19; 76) and the mean of the participants’ responses was included in the study as the participants' aesthetic ratings.

### Measures and Procedure

**Figure 5.**
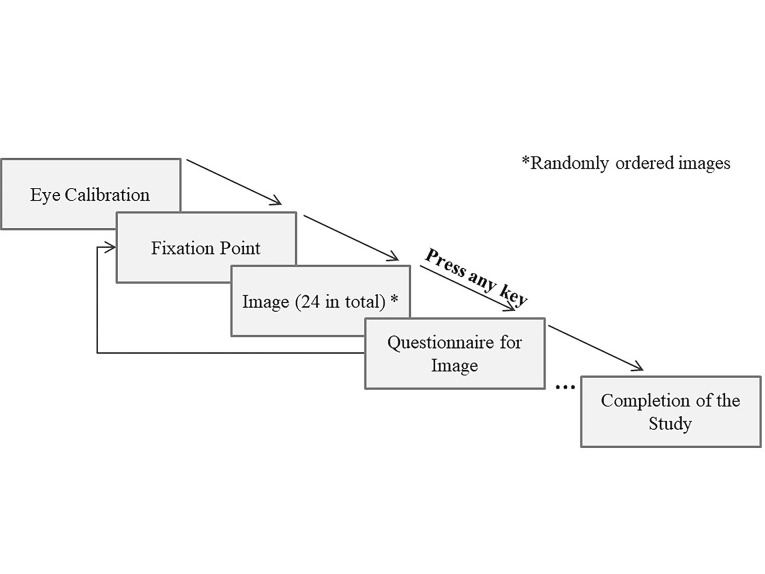
The procedure for collecting data

The experiment was conducted in The Research Focus Empirical Visual Aesthetics Lab at the University of Vienna. Eye movement data were collected during participants' exposure to the stimuli. Each participant’s eye movements were recorded using an eye tracker (EyeLink 1000; SR-Research Ltd., Mississauga, Ontario, Canada) with a monocular sampling rate of 250. The stimuli drawings displayed on a 22-inch screen (resolution 1028x1024). Participants were positioned with a 60-centimeter distance from the screen. A chin and forehead rest were used to support the head and prevent head movement. There was no time limitation during the experiment. The study phases are described below (see Figure 5).

(i) The participant’s dominant eye was identified, and eye calibrations were performed.
(ii) The fixation point was used as a focal point to facilitate eye calibration for each image. Participants directed their gaze to the fixation point prior to each manipulated stimulus and gained visual access to the images on the screen by fixating on this point.
(iii) Participants completed four practice trials in which they were familiarized with the task.
(iv) After concluding the trial tests, participants were automatically directed to the main study. Participants were free to explore the stimuli on the screen at their own pace, during which their eye movements were recorded. Once they felt ready, they were prompted to press any key on the keyboard to access the questionnaire related to the presented stimulus.
(v) Participants answered the questionnaire in the survey verbally, and no eye movements were recorded during this process.

After verbally answering the questions about the manipulated stimuli, they pressed any key on the keyboard to see the screen with the fixation point of the next stimuli. The stimuli were randomly ordered by EyeLink 1000 Experiment Builder. The study continued in this way for 24 images.

## Results

### Validation of Complexity Levels

As mentioned above, an analysis was carried out to explore whether the complexity manipulated by the experts (architects) was valid in relation to the perceived complexity of the participants. We found a positive correlation between manipulated complexity ratings and participants’ perceived complexity ratings. There were highly significant correlations for both façades manipulated with the similarity principle and those manipulated with the proximity principle, *r* = 0.95, *p* < .001 and *r* = 0.92, *p* < .001, respectively. Thus, drawings were generally perceived as manipulated in terms of complexity.

### The Influence of Gestalt Principles and Complexity on Aesthetic Evaluation

**Figure 6.**
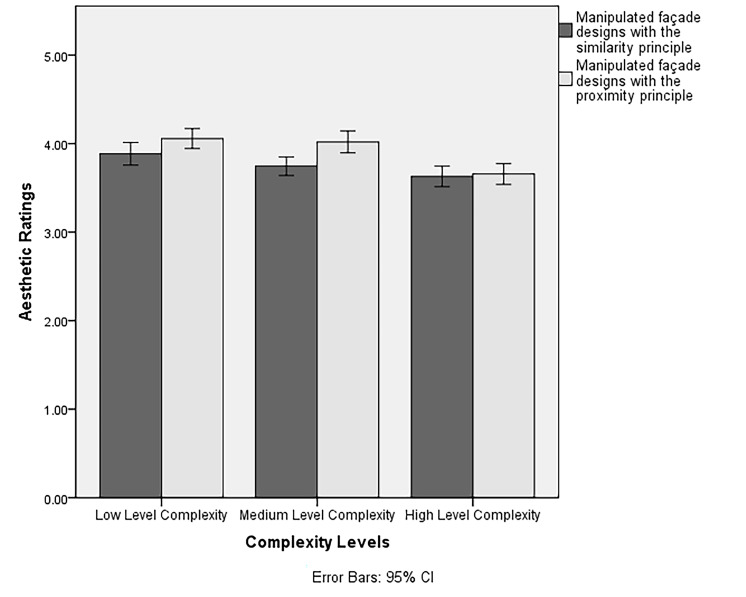
The interaction of Gestalt principles and complexity levels on participants’ aesthetic ratings

As mentioned earlier, the questionnaire was used to examine participants' aesthetic evaluations of building façade drawings. To examine the effects of Complexity Levels (3 levels) and Gestalt principles (2 levels) on participants’ aesthetic evaluation, a two-way Repeated Measures ANOVA was conducted. There was a significant main effect of level of complexity on participants’ aesthetic ratings (*F*(2,156) = 37.98, *p* < .001, *ηp^2^* = .33). In line with our Hypothesis 1a (i.e., façade compositions rated more aesthetically would have a negative correlation with complexity), a post-hoc pairwise comparison with a Bonferroni adjustment indicated that the low complexity façade compositions (*M* = 3.97, *SD* = .06) received higher aesthetic ratings than the medium (*M* = 3.89, *SD* = .05) and high complexity ones (*M* = 3.64, *SD* = .06). There was a negative linear relationship between participants’ aesthetic ratings and complexity level (see Figure 6). As a result, we could confirm Hypothesis 1a.

Moreover, there was a significant main effect of level of Gestalt principles on participants’ aesthetic ratings (*F*(1,78) = 12.57, *p* = .001, *ηp* *^2^* = .14). In line with our Hypothesis 1b (i.e., façade compositions designed with the manipulation of the similarity principle would have lower aesthetic ratings than the ones designed with the manipulation of the proximity principle), a post-hoc pairwise comparison with a Bonferroni adjustment indicated that façades designed with the manipulation of the similarity principle (*M* = 3.75, *SD* = .05) were rated aesthetically lower than the ones designed with the manipulation of the proximity principle (*M* = 3.91, *SD* = .05). Therefore, Hypothesis 1b could be confirmed. Overall, the results indicated a negative linear relationship between aesthetic ratings and complexity levels across selected Gestalt principles (see Figure 6).

### The Influence of Gestalt Principles and Complexity on Eye Movements

With respect to visual attention, we included the average fixation duration, number of fixations and viewing duration as eye tracking metrics (16; 46; 60; 79). Regarding the second aim of the study, to examine the effects of Complexity Levels (3 levels) and Gestalt principles (2 levels) on participants’ objective evaluations, again a two-way Repeated Measures ANOVA was conducted. In line with our Hypothesis 2a (i.e., participants would have the lowest fixation duration scores at high complexity levels), there was a significant main effect of level of complexity on participants’ average fixation duration scores (*F*(2,156) = 7.40, *p* = .01, *ηp* *^2^* = .09). A post-hoc pairwise comparison with a Bonferroni adjustment indicated that participants’ average fixation duration scores at high complexity level (*M* = 249.98, *SD* = 4.86) were significantly lower than medium (*M* = 270.60, *SD* = 7.23) and low complexity levels (*M* = 262.41, *SD* = 6.67). In line with our Hypothesis 2b (i.e., participants would have the highest number of fixations at high complexity levels), there was a significant main effect of level of complexity on participants’ number of fixations (*F*(2,156) = 17.63, *p* < .001, *ηp ^2^* = .18). A post-hoc pairwise comparisons with a Bonferroni adjustment indicated that participants’ number of fixations at high complexity level (*M* = 36.64, *SD* = 2.25) were significantly higher than medium (*M* = 33.91, *SD* = 2.20) and low complexity levels (*M* = 31.02, *SD* = 1.75). In line with our Hypothesis 2c (i.e., participants would have the highest total viewing duration scores at high complexity levels), there was a significant main effect of level of complexity on participants’ total viewing duration (*F*(2,156) = 4.19, *p* = .02, *ηp ^2^* = .05). A post-hoc pairwise comparisons with a Bonferroni adjustment indicated that participants’ total viewing duration scores at medium (*M* = 41103.64, *SD* = 1350.48) and high complexity levels (*M* = 40796.18, *SD* = 1345.01) were significantly higher than low level complexity level (*M* = 39565.08, *SD* = 1345.16). However, there was no significant difference between participants’ total viewing duration scores at medium and high level complexity. The analyses indicated that hypotheses 2a, 2b could be confirmed while Hypothesis 2c had to be rejected.

**Figure 7.**
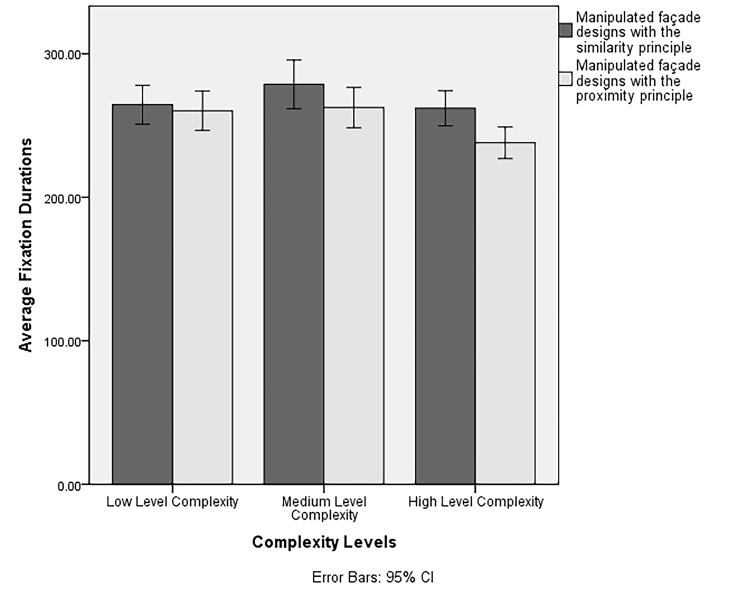
The interaction of Gestalt principles and complexity levels on participants’ average fixation duration

There was a significant interaction between complexity levels and Gestalt principles on participants’ average fixation duration (*F*(2,156) = 3.45, *p* = .03, *ηp* *^2^* = .04). A significant interaction of complexity levels and Gestalt principles was observed in terms of average fixation duration scores at the high complexity level, where participants had the lowest aesthetic ratings. A post-hoc pairwise comparison with a Bonferroni adjustment indicated that participants’ average fixation duration scores were lower for stimuli manipulated with the principle of proximity (*M* = 237.98, *SD* = 5.54) compared to those designed with the manipulation of the similarity principle (*M* = 261.98, *SD* = 6.17) at high complexity levels. An inverted U-shaped relationship was observed between the average fixation duration scores and the level of complexity for both the manipulation of the principles of similarity and proximity (see Figure 7).

**Figure 8.**
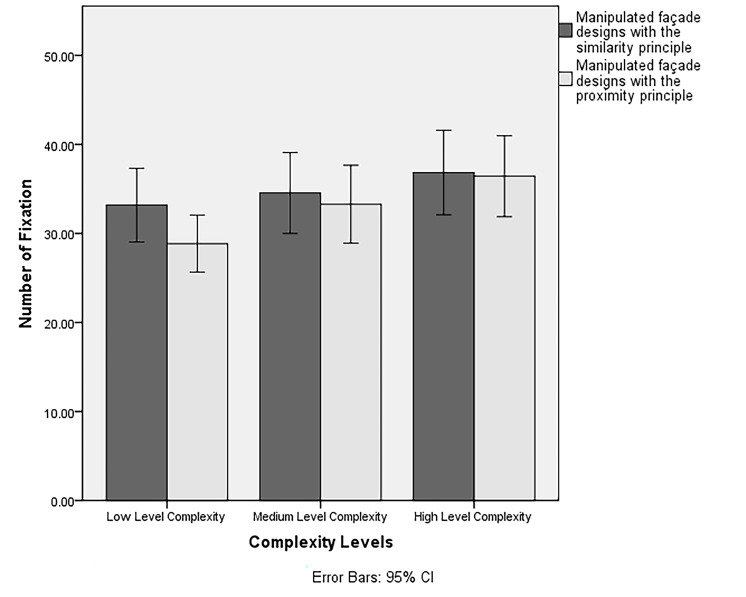
The interaction of Gestalt principles and complexity levels on participants’ number of fixations

There was a significant interaction between complexity levels and Gestalt principles on participants’ number of fixations (*F*(2,156) = 3.86, *p* = .02, *ηp* *^2^* = .05). A significant interaction of complexity levels and Gestalt principles was observed in terms of number of fixations at the low complexity level, where participants reported highest aesthetic ratings. A post-hoc pairwise comparison with a Bonferroni adjustment indicated that the participants’ number of fixations in low complexity levels were higher for façades designed with the manipulation of the similarity principle (*M* = 33.18, *SD* = 2.09) compared to those designed with the manipulation of the proximity principle (*M* = 28.86, *SD* = 1.61). A positive linear relationship was observed between the number of fixations and the level of complexity for both the principles of similarity and proximity (see Figure 8).

**Figure 9.**
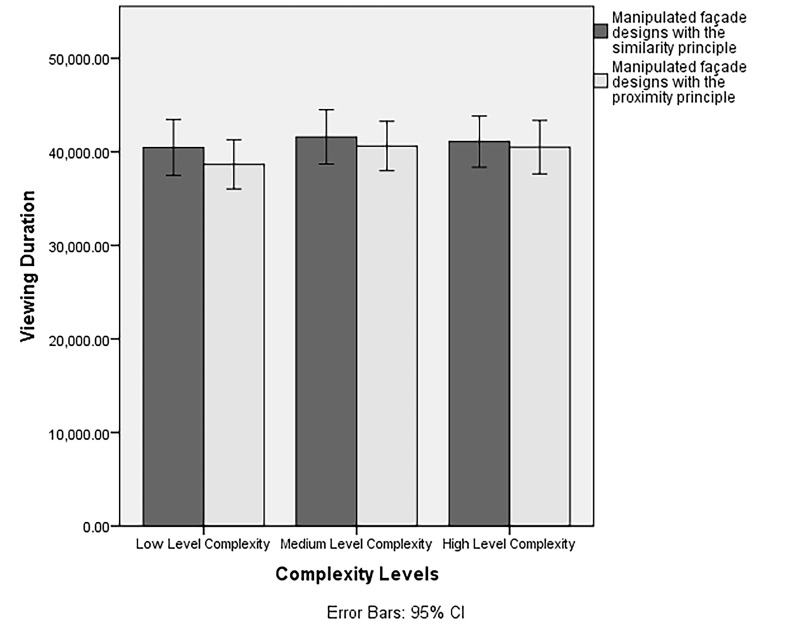
The interaction of Gestalt principles and complexity levels on participant’ total viewing duration

The interaction of complexity levels and Gestalt principles was not observed in total viewing duration results of the participants (*F*(6,468) = .73, *p* = .50, *ηp* *^2^* = .009). An inverted U-shaped relationship was observed between the total viewing duration and the level of complexity for both the principles of similarity and proximity (see Figure 9).

### The Influence of Gestalt Principles and Complexity on Participants’ Visual Attention

**Figure 10.**
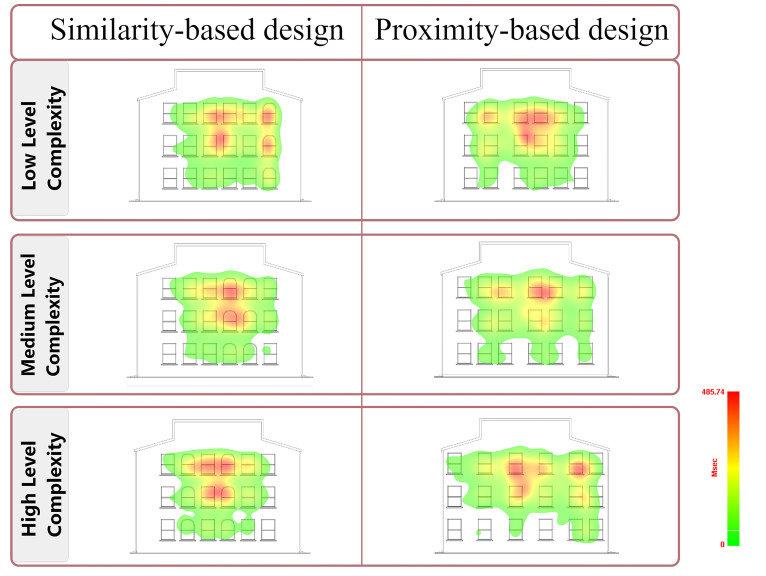
Heat maps illustrating visual attention patterns for building façade drawings

Heat maps from eye movement data model participants’ visual attention within design compositions (6). They provide an alternative visualization for aggregate fixation data (23), using a color spectrum where red indicates higher attention area. In the present study, heat maps obtained from vision fixations were utilized to identify users’ points of interest in façade compositions (see Figure 10). As the complexity level of building façade designs increased and aesthetic evaluation ratings decreased in parallel, the participants’ scanning paths became narrower. While at low complexity, attention was observed over almost the entire façade; as complexity increased, attention shifted to the area where the manipulated Gestalt principles were located, resulting in a narrower scanning area of the architectural façade. For façades where the similarity principle was manipulated, attention was directed towards differences in the shape of window frames, while for façades where the proximity principle was manipulated, attention was directed towards the distances between window frames.

## Discussion

### The Influence of Gestalt Principles and Complexity on Aesthetic Evaluation

The influence of Gestalt principles and levels of complexity on aesthetic evaluations were investigated as the first aim of the study. The results regarding the relationship between manipulated and perceived complexity showed that participants perceived the complexity levels of the drawings as intended. These findings underscore the accuracy and reliability of the experimental procedures. The results indicated that the level of complexity had a statistically significant effect on aesthetic ratings, and that the low complexity façade compositions received higher aesthetic ratings than the medium and high complexity ones. This finding is consistent with previous research (12; 27; 51), in which the level of complexity had a negative linear relationship with participants' aesthetic ratings. This suggests a preference for simpler façade compositions compared to more complex designs. In addition, façades designed with the manipulation of the proximity principle received higher aesthetic ratings than those designed with the manipulation of the similarity principle. Beyond those findings, this study aimed to bridge theoretical and empirical perspectives in the field of architectural aesthetics and may be a first step toward the exploration of how the interaction of Gestalt principles and levels of complexity influence participants’ aesthetic ratings. The results revealed that there was a negative linear association between aesthetic ratings and levels of complexity for all façade compositions manipulated with respect to the selected Gestalt principles. This would imply that — with or without the consideration of specific Gestalt principles — participants’ aesthetic ratings tend to decrease with increasing levels of architectural design complexity.

### The Influence of Gestalt Principles and Complexity on Eye Movements

Regarding the second aim of the study, higher number of fixations and shorter fixation durations were reported in participants’ eye movement data when exposed to a high complexity architectural façade, which is consistent with previous findings (13; 29; 31; 35). Participants exhibited shorter fixation durations and higher number of fixations at the high complexity level, suggesting a fast and broad visual exploration strategy in response to complex stimuli. In more detail, façades designed with the manipulation of the similarity principle received lower aesthetic ratings, demanded more time, elicited higher number of fixations, and resulted in longer fixation durations. In contrast, façades designed with the manipulation of the proximity principle received higher aesthetic ratings, demanded less time, elicited lower number of fixations, and resulted in shorter fixation durations at all complexity levels. The observed differences in fixation patterns between designs manipulated with similarity and proximity principles may reflect underlying perceptual processing strategies. Designs that use the principle of similarity increase visual coherence by grouping elements according to common characteristics, such as color or shape, and encourage viewers to process them collectively (70). Façades designed by similarity may impose a higher cognitive load due to uniformity or repeated patterns, and require more effort to process and differentiate elements. In contrast, the proximity principle involves placing elements close together for unity and organization; it aids a quick understanding of the layout, but does not mandate in-depth exploration of individual elements. Since the design elements are perceived as harmonious wholes, it may lead to faster aesthetic judgements and less cognitive load for the participants (58).

Furthermore, the medium complexity level for façade compositions resulted in a significantly higher total viewing duration compared to the high complexity level. This suggests that, contrary to our hypothesis, participants spent less viewing duration for highly complex architectural façades. The reason for this might be that individuals tend to spend more time on stimuli of moderate complexity because they may be perceived as more interesting and easier to process (32), and this is in line with the principles of cognitive load theory, which suggests that individuals may preferentially allocate cognitive resources to stimuli that are neither too simple nor too complex (68). Therefore, the observed pattern of viewing duration may reflect participants' tendency to engage more deeply with moderately complex stimuli, thus highlighting the importance of considering levels of complexity in architectural design to optimize viewer engagement and aesthetic experience.

Overall, in our study, the combination of questionnaire and eye-tracking data allowed us to obtain a more comprehensive and in-depth perspective on aesthetic judgement. We used questionnaire data to measure participants’ consciously expressed aesthetic preferences and subjective responses, but this has limitations; it may not accurately reflect participants' visual processing. Therefore, in our study, we used eye tracking devices to directly explore visual attention and processing processes. This combined approach provided an opportunity to understand participants' visual processing processes in conjunction with their reported subjective responses. The current study may contribute to our understanding of the relationship between Gestalt principles, complexity levels, and eye metrics in architectural design. The detailed patterns noted within the study would substantiate that features of organizational elements and complexity have to be considered for the design of architectural elements to be aesthetic. The findings of the present study within an architectural context may lead the way to further interdisciplinary research that intersects cognitive psychology, aesthetics, and design that may contribute to the body of knowledge regarding how individuals engage with and perceive their built environment.

### Conclusions and Limitations

The present study contributes to the existing literature by providing empirical evidence on the dynamic relationship between Gestalt principles, levels of complexity and aesthetic evaluations of façade designs. Our findings highlight the following points.

(i) Participants seemed to prefer simpler façade compositions than more complex ones, hence implying that there may be a negative linear relationship between complexity and aesthetic ratings.
(ii) As the level of complexity increased, the visual attention of the participants exhibited narrow scan paths on the façades.
(iii) Furthermore, proximity was also found to be a key determinant of aesthetic preference, resulting in higher ratings compared to similarity.
(iv) Eye tracking data revealed distinct patterns of visual exploration characterized by shorter fixation durations and a higher number of fixations for designs with high complexity.
(v) Façades designed with the manipulation of the proximity principle received higher aesthetic ratings, demanded less time, elicited lower number of fixations, and resulted in shorter fixation durations. Conversely, façades designed with the manipulation of the similarity principle received lower aesthetic ratings, demanded more time, elicited higher number of fixations, and resulted in longer fixation durations.

These findings highlight the importance of considering both organizational elements and complexity in architectural aesthetics. However, certain limitations should be acknowledged. First, the study was conducted with two-dimensional architectural façade drawings without any detailed elements, which may restrict the generalizability of the results. Future research could explore whether using more complex two- or three-dimensional architectural façade drawings or interactive images would influence the findings. Second, since our study involved participants with Austrian citizenship, and façades inspired by local buildings, it would be beneficial for future studies to examine the role of familiarity and cultural context on our results.

Despite those limitations, the present study has several strengths: Firstly, while perception studies often rely solely on the subjective evaluations of participants, our study considered both subjective and objective assessments. The integration of the eye-tracking device into the study enabled the analysis of the participants’ unconscious brain activity as part of the perception studies. Although Gestalt is an important theory in architecture, the relationship between complexity and Gestalt principles remains an underexplored area in the literature. The results of the present study could prove valuable to researchers examining the relationship between evaluation and complexity, as well as to professionals involved in the design and organization of architectural façades.

### Ethics and Conflict of Interest

The author(s) declare(s) that the contents of the article are in agreement with the ethics described in http://biblio.unibe.ch/portale/elibrary/BOP/jemr/ethics.html and that there is no conflict of interest regarding the publication of this paper.

### Acknowledgements

This study was funded by the International Research Fellowship Program (2214-A) from The Scientific and Technological Research Council of Türkiye and was conducted as part of the first author’s doctoral thesis, under the supervision of the third author. We would like to express our gratitude to all the researchers in the Art & Research on Transformations of Individuals and Societies (ARTIS) and The Research Focus Empirical Visual Aesthetics (EVAlab) group for generously allowing us to use their laboratory facilities during the data collection phase of the study. Additionally, we extend our sincere appreciation to Prof. Helmut Leder and Dr. Cengiz Acartürk for their invaluable guidance and expertise in our research endeavors.
